# Do we feel colours? A systematic review of 128 years of psychological research linking colours and emotions

**DOI:** 10.3758/s13423-024-02615-z

**Published:** 2025-01-13

**Authors:** Domicele Jonauskaite, Christine Mohr

**Affiliations:** Institute of Psychology, University of Lausanne, Lausanne, Switzerland

**Keywords:** Colour, Affect, Emotion, Perception, Association, Preferences, Cross-cultural

## Abstract

Colour is an integral part of natural and constructed environments. For many, it also has an aesthetic appeal, with some colours being more pleasant than others. Moreover, humans seem to systematically and reliably associate colours with emotions, such as yellow with joy, black with sadness, light colours with positive and dark colours with negative emotions. To systematise such colour–emotion correspondences, we identified 132 relevant peer-reviewed articles published in English between 1895 and 2022. These articles covered a total of 42,266 participants from 64 different countries. We found that all basic colour categories had systematic correspondences with affective dimensions (valence, arousal, power) as well as with discrete affective terms (e.g., *love*, *happy*, *sad, bored*). Most correspondences were many-to-many, with systematic effects driven by lightness, saturation, and hue (‘colour temperature’). More specifically, (i) LIGHT and DARK colours were associated with positive and negative emotions, respectively; (ii) RED with empowering, high arousal positive and negative emotions; (iii) YELLOW and ORANGE with positive, high arousal emotions; (iv) BLUE, GREEN, GREEN–BLUE, and WHITE with positive, low arousal emotions; (v) PINK with positive emotions; (vi) PURPLE with empowering emotions; (vii) GREY with negative, low arousal emotions; and (viii) BLACK with negative, high arousal emotions. Shared communication needs might explain these consistencies across studies, making colour an excellent medium for communication of emotion. As most colour–emotion correspondences were tested on an abstract level (i.e., associations), it remains to be seen whether such correspondences translate to the impact of colour on experienced emotions and specific contexts.

**Supplementary Information:**

The online version contains supplementary material available at 10.3758/s13423-024-02615-z.

## Introduction

*Colour not only pleases by its thousand delicate hues and harmonious gradations, but serves in nature. . . . Every passion and affection of the mind has its appropriate tint; and colouring, if properly adapted, lends its aid, with powerful effect, in the just discrimination and forcible expression of them; it heightens joy, warms love, inflames anger, deepens sadness, and adds coldness to the cheek of death itself.*

– John Opie, Cornish historical and portrait painter, 1807, Lecture IV, p. 141.

The public is interested in psychological and affective consequences of colour. Widely shared opinion holds that exposing oneself to certain colours influences one’s mood, improves well-being, or even heals.^1^ It is thus no surprise that colour is an ever-thriving economic sector with annual revenues of paint manufacturers counted in billions of dollars.^2^ Industries not only spend money on pigment and paint research, development, and production, but also on colour consultancy, textiles, interior and exterior colour design, marketing, chromotherapy, and so on. Then, claims are made about the impact of colour on one’s psychological functioning, including emotions. Although the popular media and the public sector suggest that affective connotations of colour are well-established, scientific research is only starting to answer some fundamental questions (for reviews, see Elliot, 2015, 2019; Elliot et al., 2015; Jonauskaite et al., 2025; Mohr et al., 2018; Palmer, Schloss & Sammartino, 2013). Here, we particularly focus on colour–emotion correspondences, with the goal to establish whether such correspondences are systematic across the peer-reviewed scientific studies.

Understanding how colours link to emotions has a long history (for an overview of early studies on the role of colour on human psychological functioning, see Elliot, 2019). Already Aristotle (384–322 BCE) wrote that colours have affective powers (see Fiecconi, 2020). Goethe (1810/1970) considered yellow to be agreeable and gladdening, red to convey an impression of gravity and dignity, and blue to connote excitement or repose. Such and similar beliefs are perpetuated in popular media outlets and professional settings by designers, architects, marketing, and health specialists. Scientifically, the experimental investigations into colour–emotion correspondences have been ongoing for over a century (see early studies reviewed in Ball, 1965; Dorcus, 1926; Norman & Scott, 1952). Despite these empirical studies, there is a lack of reviews that would systematise the outcome of individual studies.

To this end, we conducted the most up-to-date comprehensive systematic review on the links between colours and emotions. We considered empirical peer-reviewed articles in English, published between the end of the nineteenth century until the end of 2022. For the literature search, we used a wide range of approaches, always focussing on colour–emotion correspondences in adult populations. Regarding colour (see Box 1), we included studies working with perceptual representations of colour (e.g., defining them in terms of perceptual dimensions such as hue, chroma, lightness; Fairchild, 2013, 2015; Hunt & Pointer, 2011), and those working with conceptual representation of colour (i.e., colour terms). Regarding emotion (see Box 2), we included studies which operationalised emotion in diverse ways, whether using emotion words, emotion expressions, or felt emotions (Scherer, 2005). Consequently, studies could be separated into those working with affective dimensions (e.g., valence) and those working with discrete emotion terms (e.g., fear; Fontaine et al., 2007). We did not consider wider affective phenomena (see Box 2), such as colour preferences, cross-modal correspondences, the impact of colour on cognition, or colour–emotion links in any specific contexts (see reviews on these topics in Aslam, 2006; Elliot, 2015; Elliot & Maier, 2014; Maule et al., 2023; Palmer, Schloss & Sammartino, 2013; Spence, 2011; Thorstenson, 2018; Westland et al., 2017; Zellner, 2013). We prepared this overview following The Preferred Reporting Items for Systematic Reviews and Meta-Analyses (PRISMA 2020) guidelines (Haddaway et al., 2022; Page et al., 2021).

**Box 1. Understanding colour**

**Table:** 

***Colour perception.*** Perceived colours can be defined on a three-dimensional space of hue, saturation (chroma), and lightness (brightness; Hunt & Pointer, 2011). **Hue** is a perceptual attribute, according to which an area appears similar to one, or a combination of, the perceived colours—red, green, blue, yellow (Fairchild, 2013). **Lightness** describes how light or dark a colour is and varies on the black–white axis with shades of grey in between. A related concept of **brightness** describes how bright or dim a colour is (see further, Gilchrist, 2007). **Chroma** defines colour purity and varies on the grey–vivid axis (Valberg, 2005). Chroma is also related to **saturation**, defining the degree of colour purity relative to its lightness
***Colour language.*** People talk about colours using colour terms, which can be subdivided into basic and nonbasic (Berlin & Kay, 1969; Kay et al., 2009; McManus, 1997; Paramei & Bimler, 2021). **Basic colour terms** are frequently used words, known to all adult native speakers of a given language (Biggam, 2012a; also see Berlin & Kay, 1969, for more formal criteria to identify basic colour terms in a language). In English, there are 11 basic colour terms—namely, *red, orange, yellow, green, blue, purple, pink, brown, grey, white,* and *black—*but languages do vary in the number of basic colour terms (e.g., Androulaki et al., 2006; Berlin & Kay, 1969; Bimler & Uusküla, 2017; Davidoff et al., 1999; Paramei, 2005)*.* Most basic colour terms refer to hue (e.g., *red*, *orange*, *yellow*) but some also qualify lightness (e.g., *pink* is light red, *brown* is dark yellow or orange) or chroma (e.g., *grey*). **Nonbasic colour terms** are less frequent and are not necessarily known to all native speakers of a given language. These terms are colour descriptors arriving in many forms, for instance, by (i) adding a qualifier to a basic colour term (e.g., *sky blue, dark green, off-white*), (ii) using specialised words (e.g., *burgundy, khaki, magenta, turquoise*), or (iii) creating new phrases (e.g., *dead leaf colour, the colour of my favourite sweater*; Biggam, 2012b)

**Box 2. Understanding emotion**

**Table:** 

***Affective phenomena.*** Emotions can be distinguished from other affective phenomena (e.g., preferences, moods, affective dispositions) using the componential approach of emotion (Scherer, 2005). Accordingly, **emotions** are rapid responses to relevant changes in one’s environment. They are short lasting but intense and have a direct impact on behaviour. **Preferences** are stable aesthetic evaluative judgement of a stimulus or an event that can take the form of liking versus disliking. Preferences are often of lower intensity than emotions and overall generate unspecific positive or negative feelings (see also Palmer & Schloss, 2010; Palmer, Schloss & Sammartino, 2013; Slovic, 1995). **Moods** are diffused affective states, characterised by more stable and more enduring subjective feelings than emotions. Examples of moods include *feeling cheerful*, *gloomy*, *upset*, *depressed*, or *buoyant*. **Affective dispositions** describe stable tendencies of a person to experience certain moods or be prone to particular reactions. Examples of affective dispositions are *nervous*, *anxious*, *irritable*, *cheerful*, or *jealous*, and in their extremes, could be extended to affective pathologies like *depression*, *anxiety*, and other mood disorders
***Emotion expression and perception.*** Humans have an ability to express emotions through their faces, voices, or bodies (for reviews, see Keltner et al., 2019; Krumhuber et al., 2023; Russell et al., 2003). One can either study encoding or decoding of affective information (Witkower & Tracy, 2019). **Encoding** refers to studying the expression of emotion (i.e., display of emotion). **Decoding** refers to interpretation of affective information expressed by others (i.e., emotion recognition)
***Emotion experience.*** Humans also experience emotion subjectively (Ballard, 2021; Carstensen et al., 2000; Craig, 2009; Panksepp et al., 2017; Reisenzein & Döring, 2009; Weidman & Tracy, 2020). When people say, “I feel good”, “I am happy”, or “I am afraid”, they are expressing feelings. It is, however, not evident how such experiences should be assessed. There are various widely used **self-report measures of emotion experience**, including the Self-Assessment Manikins (SAM; Bradley & Lang, 1994), the Positive and Negative Affect Schedule (PANAS; Watson et al., 1988), and the Geneva Emotion Wheel (GEW; Scherer, 2005; Scherer et al., 2013). When asking participants directly how they feel, one must assume that participants have a good insight into their internal experiences, which is not always the case (e.g., Demiralp et al., 2012; Hoemann et al., 2021). Then, one can also assess participants’ **psychophysiological responses**, with a hope to gain insights into their affective experiences. Researchers might consider brain imaging techniques to record neural patterns such as EEG, fMRI, or NIRS or record participants’ heart rate, skin conductance response, facial muscle activity, and breathing patterns (Kreibig, 2010; Mauss & Robinson, 2009). Psychophysiological recordings largely assess changes in autonomic arousal states (i.e., psychophysiological activation or excitement; Kreibig, 2010; Levenson, 2014; Mauss & Robinson, 2009), making exact emotion identification and specification challenging
***Emotion language***. In addition to using **distinct affective terms** such as *anger, fear, sadness, joy* (e.g.Cowen & Keltner, 2017; Darwin, 1872; Ekman & Friesen, 1971; Tracy & Randles, 2011), one can consider the relationships between the different emotion concepts and define them along **affective dimensions.** Fontaine and colleagues (2007) concluded on four principal dimensions that were most helpful in organising distinct emotion concepts in languages—namely, valence, arousal, power, and novelty (also see Osgood et al., 1957; Russell, 1980; Shaver et al., 1987). **Valence**, also called *evaluation*, *hedonic tone*, *pleasantness*, or *pleasure*, describes the degree to which an object or an event is considered positive or negative, or the affective response is considered pleasant or unpleasant (Itkes & Kron, 2019). Examples of positive emotions include *joy*, *pride*, and *relief*, and negative emotions include *anger*, *contempt*, and *disappointment*. **Arousal** has also been called *activation*. It describes the degree of excitation, often ranging from *calm* to *excited*. From our set of examples, arousing emotions would be *joy* and *anger*, while low arousing emotions would be *contempt*, *pride*, *disappointment*, and *relief*. Arousal and valence dissociate, because positive as well as negative emotions can be arousing. **Power** has also been called *potency*, *control*, or *dominance*. It describes one’s judgement of having control over a situation. For instance, a person might feel empowered by an experience and wants to do something about or with the experience. Else, a person might feel unable to take control or action. Empowering emotions would be *joy*, *anger*, *contempt*, and *pride*, while disempowering emotions would be *disappointment* and *relief*. Finally, **novelty** separates emotions based on their predictability. Examples of emotion concepts high in novelty are *surprise*, *awe*, and *astonishment*

### Method

**Fig. 1:**
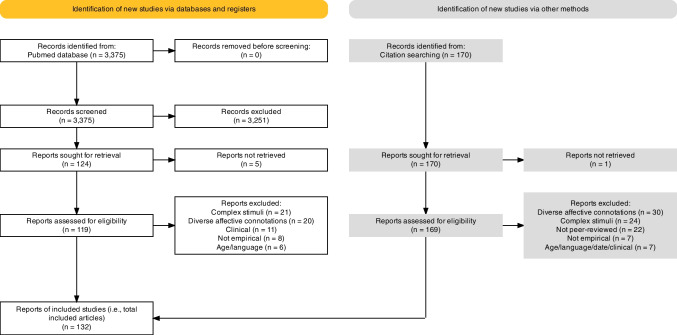
The flowchart showing the process of record screening and report selection complying with the PRISMA 2020 guidelines (Haddaway et al., 2022; Page et al., 2021)

We prepared this systematic review following The Preferred Reporting Items for Systematic Reviews and Meta-Analyses (PRISMA 2020) guidelines (Haddaway et al., 2022; Page et al., 2021). Below, we describe the process of identification of the relevant journal articles (reports), complying with the PRISMA 2020 checklist (https://prisma.shinyapps.io/checklist/)—see Fig. 1. In this section, we are using terminology in accordance with PRISMA 2020 guidelines:

- *Record*: the title and/or an abstract of a report indexed in a database or a website.
- *Report*: a journal article, a preprint, a conference abstract, or a similar document supplying information about the study. In other words, a report is the full text of the record.
- *Study*: a scientific investigation. Most reports include one study; however, some reports might include multiple studies and vice versa.

### Literature search and article selection

The goal of the literature search was to compile a comprehensive list of peer-reviewed empirical articles published on context-free colour–emotion correspondences. We conducted the literature search on PubMed database (https://pubmed.ncbi.nlm.nih.gov/) with the search terms COLO*R (to include both spelling variants—color and colour) and EMOTION, retrieving 3,375 records (see ‘Records identified from Pubmed database’ in Fig. 1).

#### Phase 1: Record screening by title

We screened 3,375 record titles, using the following criteria (see ‘Records screened’ in Fig. 1):

- *Date*: include reports published by December 2022.
- *Humans*: include only human studies; exclude animal studies.
- *Peer-review*: include only peer-reviewed reports (i.e., journal articles); exclude conference proceedings, unpublished theses, industry reports, etc.
- *Language*: include reports published in the English language; exclude full reports published in other languages.
- *Empirical investigation*: include only empirical studies; exclude reviews, meta-analyses, etc.
- *Age range*: include only reports on adult population; exclude reports focused entirely on children or elderly. If different age groups were studied, we present results on the adult population only.
- *Clinical studies*: include only healthy populations; exclude clinical samples (e.g., depression, anxiety, schizophrenia, synaesthesia) and studies with a clinical focus (e.g., healing) or using clinical tests (e.g., Rorschach test, Lüscher test)
- *Biological studies*: include only studies investigating psychological research questions; exclude studies focused on biological mechanisms of colour vision, or similar.
- *Diverse meanings of colour*: include reports where colour was used as stimulus; exclude reports where colour had other meanings, for instance, referred to skin colour.
- *Diverse affective connotations*: exclude reports of colour preferences (i.e., liking or disliking a particular colour), or reports testing colour-music, colour-odour, colour-taste, colour-temperature, etc., or reports applicable to specific contexts (e.g., food).

After the Phase 1 record screening, we kept 124 PubMed records for a closer inspection in Phase 2 (see ‘Reports assessed for eligibility’ in Fig. 1). Furthermore, we decided to conduct additional searches of records using backward and forward search of reference lists of relevant articles because we realised that multiple relevant records were not captured with the initial search. Using this technique, we identified an additional number of 169 nonduplicate records of potentially relevant reports. We also inspected those in Phase 2 (see “Records identified from citation searching” and ‘Reports assessed for eligibility’ in Fig. 1). In total, we inspected 293 reports (i.e., 124 + 169) in Phase 2.

#### Phase 2: Record screening by abstract and full text

We screened 293 reports by judging their abstracts and full texts. In addition to the Phase 1 criteria, we also applied the following criteria (see ‘Reports assessed for eligibility’ in Fig. 1, separated by database):

- *Colour combinations*: exclude reports in which colour combinations instead of discrete colours were judged (e.g., artworks, complex visual scenes, colour combinations)
- *No emotion/affect*: exclude reports in which no affective connotations were assessed (implicitly or explicitly) or where the terms were too ambiguous (e.g., measured associations with the term ‘emotion’).

After the Phase 2 record screening, we kept 132 reports to be included in the current systematic review (see Fig. 1). The 132 reports were peer-reviewed articles of empirical studies. Thus, we refer to them as articles and not as reports throughout the remaining text.

#### Data preparation and analysis

We extracted the following information from each article: authors, publication title, publication outlet, digital object identifier (DOI), studied population, number of participants in the relevant groups (e.g., only adult participants or nonclinical population), gender distribution (i.e., proportion of men), participants’ age (mean age, *SD*, and/or range of age), country of testing, information about colour and emotion variables (approach, colour system, the exact colours and emotions included, etc.), key outcome measures and key results. When articles reported several studies or collected data in several countries, we summed the number of participants and determined the gender proportion for this total sample. We used these pieces of information to synthesise the (i) key demographic information, (ii) types of methodological choices, and (iii) key results on colour–emotion correspondences. We separated the latter into results on affective dimensions (i.e., valence, arousal, and power)^3^ and discrete affective terms. We give details on statistical tests directly in the results section.

#### Transparency and openness

We adhered to the PRISMA 2020 guidelines for systematic reviews (Haddaway et al., 2022; Page et al., 2021). All data and research materials are available online (https://osf.io/g5srf). This review was not preregistered.

## Results

**Fig. 2:**
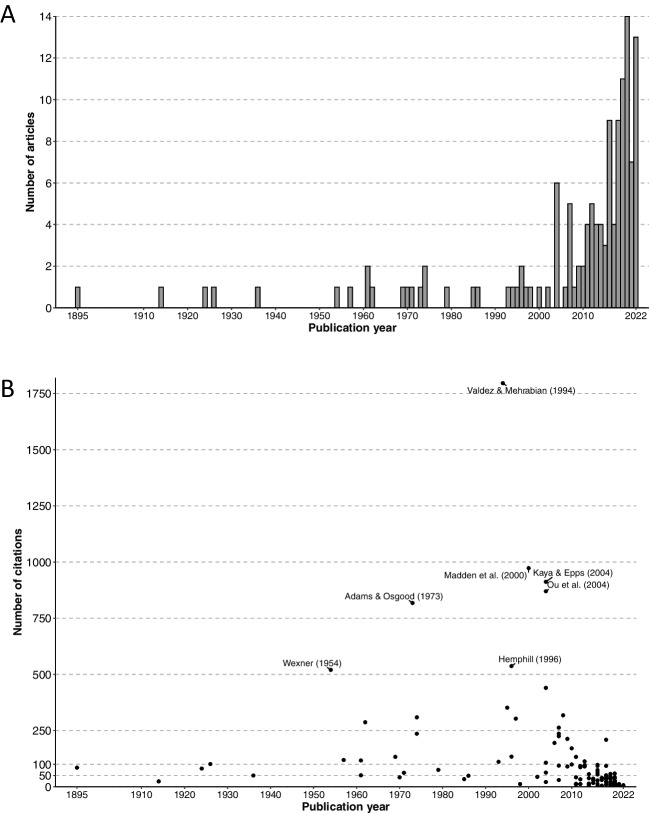
**A** The chronological order of the publication timeline of 132 articles studying colour–emotion correspondences, published between 1895 and 2022.** B** The chronological order of the number of citations received by the articles. Articles with 500 or more citations are labelled (see the interactive figure with all citations labelled here: https://www2.unil.ch/onlinepsylab/Figures/Review/Plot_citations_pub_years_interactive.html)

The first of the 132 articles appeared in 1895 and the latest in 2022, marking 128 years of scientific research on colour–emotion correspondences (see Table 1). Purely numerically, these numbers would mean that there were about 1.03 articles per year. In reality, however, most articles were published in the past two decades (see Fig. 2A) with 26 articles published between 2003 and 2012 and 78 articles published between 2013 and 2022.

**Table 1:** The list of 132 empirical articles investigating colour-affect correspondences, ordered chronologically and then alphabetically

Authors	Year	Country	*N*	Colour method: main type	Colour method: Subtype	Emotion method: Main type
Major	1895	USA	3	Visual	Colour patches	Affective words
Fernberger	1914	USA	15	Visual	Colour patches	Affective words
Nafe	1924	USA	7	Visual	Colour patches	Affective words
Dorcus	1926	USA	871	Visual	Colour patches	Affective words
Allen & Guilford	1936	USA	10	Visual	Colour patches	Affective words
Wexner	1954	USA	94	Visual	Colour patches	Affective words
Murray & Deabler	1957	USA	25	Visual	Colour patches	Affective words
Schaie	1961a	USA	20	Visual	Colour patches	Affective words
Schaie	1961b	USA	44	Visual	Colour patches	Affective words
Wright & Rainwater	1962	Germany	3,660	Visual	Colour patches	Affective words
Hogg	1969	UK	133	Visual	Colour patches	Affective words
Pecjak	1970	Multiple (8 countries)	457	Verbal	Colour terms	Affective words
Nourse & Welch	1971	USA	14	Visual	Coloured lights	Psychophysiological response
Adams & Osgood	1973	Multiple (23 countries)	920	Verbal	Colour terms	Affective words
D'Andrade & Egan	1974	Multiple (2 countries)	52	Visual	Colour patches	Affective words
Jacobs & Hustmyer	1974	USA	24	Visual	Coloured lights	Psychophysiological response
Hogg et al	1979	UK	20	Visual	Colour patches	Affective words
Kunishima & Yanase	1985	Japan	30	Visual	Colour patches	Affective words
Johnson et al	1986	Peru	18	Visual	Colour patches	Affective words
Ainsworth et al	1993	USA	45	Visual	Wall colours	Affective words
Valdez & Mehrabian	1994	USA	250	Visual	Colour patches	Affective words
Terwogt & Hoeksma	1995	The Netherlands	24	Visual	Colour patches	Affective words
Collier	1996	USA	47	Visual	Colour patches	Affective words
Hemphill	1996	Australia	40	Visual	Colour patches	Affective words
Hupka et al	1997	Multiple (5 countries)	661	Verbal	Colour terms	Affective words
Ziems et al	1998	USA	36	Visual	Colour patches	Affective state
Madden et al	2000	Multiple (7 countries)	253	Visual and verbal	Colour patches and colour terms	Affective words
Hatta et al	2002	Japan	12	Visual	Physical object colours	Affective words
Kaya & Epps	2004	USA	98	Visual	Colour patches	Affective words
Leichsenring	2004	Germany	140	Visual	Colour patches	Affective words
Meier et al	2004	USA	169	Visual	Font colour	Affective words
Ou et al	2004	Multiple (2 countries)	31	Visual	Colour patches	Affective words
Xin et al	2004a	Multiple (3 countries)	210	Visual	Colour patches	Affective words
Xin et al	2004b	Multiple (3 countries)	210	Visual	Colour patches	Affective words
Gao & Xin	2006	China (Hong Kong)	70	Visual	Colour patches	Affective words
Da Pos & Green-Armytage	2007	Australia	44	Visual	Colour patches	Facial expressions
Gao et al	2007	Multiple (7 countries)	440	Visual	Colour patches	Affective words
Manav	2007	Turkey	50	Visual	Colour patches	Affective words
Meier et al	2007	USA	185	Visual	Colour patches	Affective words
Steinvall	2007	Bank of English corpus	NA	Verbal	Colour terms	Affective words
Clarke & Costall	2008	UK	16	Verbal	Colour terms	Affective words
Moller et al	2009	USA	72	Visual	Font colour	Affective words
Soriano & Valenzuela	2009	Spain	115	Verbal	Colour terms	Affective words
Carruthers et al. (Study 2)	2010	UK	204	Visual	Colour patches	Affective words
Suk & Irtel	2010	Germany	85	Visual	Colour patches	Bodily expressions
Sakuragi & Sugiyama	2011	Japan	20	Visual	Physical object colours	Affective words
Simmons	2011	UK	116	Visual	Colour patches	Affective words
Williams et al	2011	Canada	14	Visual	Colour glasses	Affective words
Yildirim et al	2011	Turkey	290	Visual	Wall colours	Affective words
Fetterman et al	2012	USA	265	Visual	Font colour	Affective words
Joosten et al	2012	The Netherlands	51	Visual	Coloured lights	Bodily expressions
Lakens et al	2012	The Netherlands	320	Visual	Font colour and background colour	Affective words
Lechner et al	2012	Multiple (12 countries)	2,021	Visual	Colour patches	Affective words
S. Wang & Ding	2012	China	20	Visual	Colour patches	Affective words
Kuhbandner & Pekrun	2013	Germany	42	Visual	Font colour	Affective words
Lakens et al	2013	The Netherlands	205	Visual and verbal	Images with modified colour scheme	Affective words
Palmer, Schloss, Xu, et al	2013	Multiple (2 countries)	121	Visual	Colour patches	Affective words and facial expressions
Young et al	2013	USA	66	Visual	Background or clothing colour	Facial expressions
Buechner et al	2014	Germany	159	Visual	Background or clothing colour	Facial expressions
Sandford	2014	USA	106	Verbal	Colour terms	Affective words
T. Wang et al	2014	China	58	Visual and verbal	Colour patches	Affective words
Zhang et al	2014	China	48	Visual	Colour patches	Affective words
Gil & Le Bigot	2015	France	44	Visual	Background or clothing colour	Facial expressions
Koo & Kwak	2015	South Korea	17	Visual	Coloured lights	Affective words
Meier et al	2015	USA	980	Visual	Font colour	Affective words
Al-Ayash et al	2016	Australia	24	Visual	Wall colours	Affective words
Dael et al	2016	Switzerland	28	Visual	Colour patches	Bodily expressions
Gil & Le Bigot	2016	France	76	Visual	Background or clothing colour	Facial expressions
Gilbert et al	2016	USA	110	Visual	Colour patches	Affective words
Hanafy & Reham	2016	Oman	80	Unknown	NA	Affective words
Mammarella et al	2016	Italy	50	Visual	Font colour and images with modified colour scheme	Affective words
Sutton & Altarriba	2016a	USA	118	Verbal	Colour terms	Affective words
Sutton & Altarriba	2016b	USA	105	Verbal	Colour terms	Affective words
Zieliński	2016	Poland	67	Visual	Colour patches	Affective words and psychophysiological response
Barchard et al	2017	Multiple (2 countries)	366	Verbal	Colour terms	Affective words
Goodhew & Kidd	2017	Australia	25	Verbal	Colour terms	Affective words
Mentzel et al	2017	Germany	29	Visual	Font colour	Affective words
Nakajima et al	2017	Japan	20	Visual	Facial colour	Facial expressions
Hanada	2018	Japan	47	Visual	Colour patches	Affective words
Minami et al	2018	Japan	20	Visual	Facial colour	Facial expressions
Ou et al	2018	Multiple (7 countries)	658	Visual	Colour patches	Affective words
Specker & Leder	2018	Austria	30	Visual	Colour patches	Affective words
Specker et al	2018	Multiple (2 countries)	122	Visual	Colour patches	Affective words
Takahashi & Kawabata	2018	Japan	40	Visual	Colour patches	Affective words and facial expressions
Thorstenson et al	2018	USA	330	Visual	Facial colour	Affective words
Wilms & Oberfeld	2018	Germany	62	Visual	Colour patches	Bodily expressions and psychophysiological response
Young et al	2018	USA	40	Visual	Facial colour	Facial expressions
Fugate & Franco	2019	Multiple (3 countries)	150	Visual	Colour patches	Affective words
Ismael & Ploeger	2019	Germany	487	Visual	Colour patches	Affective state
Jonauskaite, Abdel-Khalek, et al	2019	Multiple (55 countries)	6,625	Verbal	Colour terms	Affective words
Jonauskaite, Althaus, et al	2019	Switzerland	96	Visual	Colour patches	Affective state
Jonauskaite, Dael, et al (study 3)	2019	Switzerland	183	Verbal	Colour terms	Affective words
Jonauskaite, Wicker, et al	2019	Multiple (4 countries)	711	Verbal	Colour terms	Affective words
Kiselnikov et al	2019	Russia	102	Verbal	Colour terms	Affective words
Kramer & Prior	2019	UK	100	Visual	Background or clothing colour	Affective words
Peromaa & Olkkonen	2019	Finland	40	Visual	Facial colour	Facial expressions
Thorstenson et al	2019	USA	195	Visual	Facial colour	Facial expressions
Cha et al	2020	China (Hong Kong)	55	Visual	Wall colours	Affective words
Chen et al	2020	Multiple (2 countries)	30	Visual	Colour patches	Affective words
Demir	2020	Turkey	929	Visual	Colour patches	Affective words
Goodhew & Kidd	2020	Australia	34	Visual	Font colour	Affective words
Güneş and Olguntürk	2020	Turkey	180	Visual	Wall colours	Facial expressions
Hu et al	2020	USA	20	Visual	Colour patches	Affective words
Jonauskaite, Abu-Akel, et al	2020	Multiple (30 countries)	4,598	Verbal	Colour terms	Affective words
Jonauskaite, Parraga, et al	2020	Switzerland	132	Visual and verbal	Colour patches	Affective words
Kawai et al	2020	Austria	145	Visual	Font colour	Affective words
Lipson-Smith et al	2021	Australia	745	Visual	Wall colours	Affective words
Ram et al	2020	Multiple (3 countries)	944	Verbal	Colour terms	Affective words
Schloss et al	2020	USA	68	Visual	Colour patches	Affective words
Tham et al	2020	Multiple (2 countries)	256	Visual	Colour patches	Affective words
Ulusoy et al	2020	Turkey	15	Visual	Colour patches	Affective words
Jonauskaite, Camenzind, et al	2021	Switzerland	130	Visual and verbal	Colour patches	Affective words
Jonauskaite, Sutton, et al	2021	English language corpus (GloVe)	NA	Verbal	Colour terms	Affective words
Lee et al	2021	South Korea	30	Visual	Coloured lights and wall colours	Affective words
Ruba et al. (Exp 1)	2021	USA	60	Visual	Background or clothing colour	Facial expressions
Saysani et al	2021	Australia	20	Verbal	Colour terms	Affective words
Winskel et al. (Exp 1 and 2)	2021	Australia	50	Visual	Font colour	Affective words
Wolf et al	2021	Germany	609	Visual	Facial colour	Facial expressions
Avery et al	2022	USA	1,059	Verbal	Colour terms	Affective words
Baniani	2022	Japan	47	Visual	Facial colour	Facial expressions
Yar Bilal et al	2022	Turkey	273	Visual	Coloured lights and wall colours	Affective words
Bower et al	2022	Australia	18	Visual	Wall colours	Bodily expressions and psychophysiological response
Kang et al	2022	South Korea	40	Visual	Facial colour	Facial expressions
Lee & Lee	2022	USA	82	Visual	Coloured lights	Affective words
Liao et al	2022	Japan	52	Visual	Facial colour and colour patches	Affective words and facial expressions
Oh & Park	2022	South Korea	24	Visual	Wall colours	Affective words and psychophysiological response
Takei & Imaizumi	2022	Japan	20	Visual	Background or clothing colour	Facial expressions
Thorstenson et al	2022	USA	374	Visual	Facial colour	Facial expressions
Bouhassoun et al	2023	France	152	Visual	Font colour	Affective words
Kawai et al	2023	Multiple (4 countries)	439	Visual	Font colour	Affective words
Uusküla et al	2023	Multiple (28 countries)	4,008	Verbal	Colour terms	Affective words
Zaikauskaite et al	2023	UK	605	Visual	Colour patches	Affective words

The number in the brackets after ‘Multiple’ indicates the number of included articles in the review. *N* = the number of participants. Articles with the publication date of 2023 were first-online published before or in 2022

On average, the articles were cited 110.7 times (*Median* = 39, *SD* = 227.3 citations). Valdez and Mehrabian’s (1994) article was on top of the list, with 1,796 citations. Seven articles had more than 500 citations, 14 articles had more than 250 citations, and 30 articles had more than 100 citations (see Fig. 2B). On the other end of the spectrum, 19 articles received five or fewer citations. Articles were published in 68 different outlets (i.e., journals and books). The most popular publication outlets (*n* articles) were *Color Research & Application* (21), *Emotion* (7), *Perceptual and Motor Skills* (7), *Frontiers in Psychology* (6), *The American Journal of Psychology* (5), and *Acta Psychologica* (5).

### Demographic data

#### Sample sizes, age, and gender

Two studies in the reported articles were based on linguistic corpora and thus did not include participants. In the remaining studies, there were 42,266 participants in total, each study including between three and 6,625 participants (*M* = 325.1, *Median* = 74, *SD* = 860.2). From these, 35 articles included more than 200 participants, 17 articles included more than 500 participants, six articles included more than 1,000 participants, and one article included more than 5,000 participants.

Fifty-nine articles did not report the mean or median age of participants and 20 articles did not report gender constitution. From the articles reporting age and/or gender information, the mean reported age of participants was 24.3 (*Median* = 22.0, *SD* = 5.56, range of mean age = 18.5–42.4). On average, 39.5% of participants were men (*Median* = 40.5%, *SD* = 20.4%), with seven articles focusing exclusively on women and three articles focusing exclusively on men. See Table 1 for further details on the demographic details of the articles and supplemental material.

#### Participant country

**Fig. 3:**
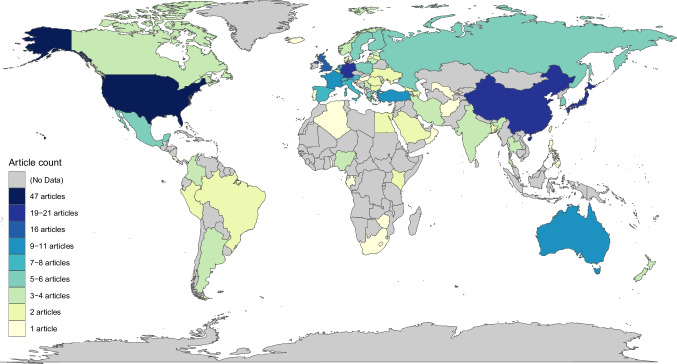
Count of articles, which included each country in their dataset, pooled across single-country and multi-country articles. Bluer and darker colours indicate a larger number of articles. (Colour figure online)

Articles that included participants (i.e., noncorpus studies; *n* = 130) largely studied participants from one country (*n* = 107). The large majority of articles studied participants from Western countries (*n* = 79), while the rest focused on non-Western countries (*n* = 28). The remaining 23 articles studied multiple countries, with the number of studied countries being between two and 55 countries (*M* = 9.4, *Median* = 4, *SD* = 12.9 countries). Four multicountry articles particularly stood out, including 23 countries, 28 countries, 30 countries, and 55 countries in their datasets (see Table 1). Apart from these four articles, the range of studied countries in the remaining multicountry articles was between two and 12 countries (*M* = 4.2, *Median* = 3, *SD* = 2.78 countries). Overall, 63 different countries were studied across all multicountry articles. Across all articles (i.e., single- and multicountry articles), 64 different countries were studied, with Oman included once in a single-country article. As shown in Fig. 3, the most frequently studied countries were USA (*n* = 47), China (*n* = 21), Germany (*n* = 19), Japan (*n* = 19), UK (*n* = 16), Australia (*n* = 11), and Turkey (*n* = 10).

### Methodological approaches

#### Studying colour

We found that 105 articles (79.5%) presented colours visually, 21 articles (15.9%) presented colours verbally (i.e., colour terms), five articles (3.8%) used both visual and verbal methods, and one article (0.8%) did not report enough information to determine the colour presentation mode. Researchers used diverse types of visually presented colours, with the most frequent choices being colour patches, font colours, and facial colours (see Table 2).

**Table 2:** Colour presentation methods used in the reviewed articles

Colour presentation mode	Subtype of colour presentation mode	*n* articles	% from total
Visual presentation	All together	110	83.3
	Colour patches (colour squares)	61	46.2
	Font colours	12	9.1
	Facial colours	11	8.3
	Wall colours	10	7.6
	Background or clothing colours	8	6.1
	Coloured lights	7	5.3
	Colours of physical objects	2	1.5
	Images with modified colour scheme	2	1.5
	Colour glasses	1	0.8
Verbal presentation	Colour terms	25	19.7

*Note*. Five articles used both visual and verbal colour presentation modes; five articles used two different subtypes of colour presentation modes, and one article did not report sufficient amount of information to decipher the method they used. This explains why the line *All together* does not represent a simple sum of the different subtypes of colour presentation modes. Nonetheless, we took 132 as the total number of articles to calculate the percentages. See supplemental material and Table 1 to see which articles used which method

When articles used colour terms (*n* = 25), they used on average 10.5 colour terms (*Median* = 11, *SD* = 9.6). Two studies used an unrestricted selection of colour terms by allowing their participants to freely write colour terms that came to their minds. When studies used a visual colour presentation mode (*n* = 110), researchers used on average 32.4 shades of colour (*Median* = 8, *SD* = 58.6), indicating a large variability between articles. Ten articles used an unrestricted sample of colours. They either allowed participants to choose colours with a colour picker which gives access to all colours a computer screen can produce or asked participants to manipulate colours on chromatic dimensions (usually *CIE Lab* a* + redness, a*- greenness, b* + yellowness, and b*- bluishness). One article did not report sufficient information to judge the number of colours.

Articles specified colours using diverse colour systems, with CIE (International Commission on Illumination) and RGB systems being the most frequent. About half of the articles (58.7%) used perceptually uniform colour spaces, about a third (27.5%) used perceptually non-uniform colour spaces, and the remaining 13.8% of articles did not specify the colour space they used (see Table 3).

**Table 3:** Colour models used in the reviewed articles

Perceptually uniform colour spaces			Perceptually nonuniform colour spaces		
Colour space	*n* articles	% from total	Colour space	*n* articles	% from total
CIE *Lab*, CIE *LCh*, CIE *Luv*	32	29.4	RGB, HSL, or HSB	25	22.9
Munsell Color System	25	22.9	Milton Bradley or Stoelting coloured papers or Liquitex paint company	5	4.6
Natural Color System	7	6.4	Unknown	16	14.5
Total	64	58.7	Total	46	41.8

Colour systems which can be converted between each other using arithmetic conversations were grouped under the same umbrella term. *CIE* = The International Commission on Illumination (abbreviation used for its French name *Commission internationale de l'éclairage*); RGB = Red, Green, Blue; HSL = Hue, Saturation, Lightness; HSB = Hue, Saturation, Brightness. Total = 110 articles including visual colours. See supplemental materials to see which articles used which method

### Studying emotion

There were 125 articles (94.7%) using one method and seven articles (5.3%) using two methods to assess emotion, either as a stimulus or as an outcome variable. The most popular method involved affective words with 105 articles choosing this method. Other methods were less popular: facial expressions, psychophysiological responses, bodily expressions, or affective states (see Table 4).

**Table 4:** Emotion assessment methods used in the reviewed articles

Emotion assessment method	Subtypes of emotion assessment method	*n* articles	% from total
Affective words	All subtypes together	105	79.5
	Dimensional approach (i.e., semantic differentials, valence, arousal, power)	62	47.0
	Discrete affective terms	32	24.2
	Other (i.e., questionnaires and an unrestricted range)	11	8.3
Facial affective expressions	All subtypes together	19	14.4
	Human faces	16	12.1
	Pictograms of faces (e.g., emoticons)	3	2.3
Psychophysiological responses	Heart rate, skin conductance response, etc	6	4.5
Bodily affective expressions	All subtypes together	6	4.5
	Human bodies	1	0.8
	Pictograms of bodies (e.g., SAM)	5	3.8
Affective states	Induced mood	3	2.3

Seven articles used two types of emotion assessment methods and some articles used two subtypes of the same emotion assessment method. This explains why the total count of articles is above 132. Nonetheless, we took 132 as the total number of articles to calculate the percentages. See supplemental materials and Table 1 to see which articles used which method

Some of these main types of emotion assessment methods could be further categorised into subtypes. For instance, a third of the articles using affective words also worked with discrete affective terms (*n* = 32), like *love*, *joy*, *sadness*, and so on. Articles used between two and 135 discrete affective terms, with an average of 18.1 terms (*Median* = 14, *SD* = 22.1). Strictly speaking, not all of these terms referred to emotions, but all had affective loadings (e.g., *death, aggression, relaxation*).

Furthermore, even more articles using affective words employed the dimensional approach (*n* = 62). Among them, the most popular method was to measure valence, arousal, and/or power dimensions, used in 39 articles. From these articles, most assessed only valence (*n* = 23 articles), for instance, by asking participants to rate how positive or negative a colour patch was. Another popular dimensional approach was semantic differential scales (*n* = 26), such as *active*–*passive*, *good–bad*, *hot–cold*, and so on. Articles used between 2 and 47 semantic differential scales, with the average being 14.3 scales (*Median* = 12.0, *SD* = 10.4). Not all semantic scales measured emotion and some of the scales closely matched the affective dimensions of valence, arousal, and power. Thus, three articles chose to cluster their responses, obtained on semantic differential scales, along valence, arousal, and/or power dimensions.

### Key results on colour–emotion correspondences

Most articles (*n* = 79, 59.8%) assessed colour–emotion correspondences using explicit methods. In other words, they asked participants to associate colours and emotions directly. In these cases, the goal of the experiment was evident to the participants. About a third of articles (*n* = 48, 36.4%) used implicit methods (e.g., implicit association test) to assess colour–emotion correspondences, making it more difficult to guess the goal of the experiment. Finally, five articles (3.8%) used both implicit and explicit methods.

We included results on chromatic colour categories (RED, ORANGE, YELLOW, GREEN, GREEN–BLUE, BLUE, PURPLE, PINK, BROWN) and achromatic colour categories (WHITE, GREY, BLACK). Although the GREEN–BLUE category is not basic, we added it to the 11 basic colour categories because (i) it has been used in systematic and extensive global studies on colour–emotion correspondences (e.g., Jonauskaite, Abu-Akel, et al., 2020; Kaya & Epps, 2004), (ii) the colour term *turquoise* has augmented the British English basic colour term lexicon (Mylonas & MacDonald, 2016), and (iii) the colour term *teal* is an emerging basic colour term in American English (Lindsey & Brown, 2014). We further included results on lightness/brightness (LIGHT/BRIGHT, DARK) and chroma/saturation (SATURATED, DESATURATED). Since articles used vastly different stimuli, there likely was a large variability in the colour samples shown for each colour category. Not all articles studied all the colour categories (see Tables 5, 6, 7, 8, 9 and 10 on the number of articles studying each colour category).

**Table 5:** The number of articles which studied a correspondence between a given colour category and each affective dimension and direction of the results

Colour category	Valence		Arousal		Power		Discrete affective terms
	*n* of articles	Direction	*n* of articles	Direction	*n* of articles	Direction	*n* of articles
RED	40	Inconclusive	24	High	14	High	54
ORANGE	19	Inconclusive	13	Inconclusive	7	Inconclusive	17
YELLOW	32	Positive	22	High	12	Inconclusive	41
GREEN	36	Positive	23	Low	13	Inconclusive	35
GREEN–BLUE	4	Positive	3	Inconclusive	3	Inconclusive	8
BLUE	39	Positive	27	Low	14	Low	40
PURPLE	24	Inconclusive	16	Inconclusive	11	High	20
PINK	8	Positive	3	Inconclusive	2	Inconclusive	16
BROWN	13	Negative	9	Inconclusive	6	Inconclusive	13
WHITE	24	Positive	15	Low	9	Inconclusive	23
GREY	22	Negative	13	Low	8	Low	20
BLACK	22	Negative	14	Inconclusive	10	High	28
LIGHT/BRIGHT	19	Positive	6	Inconclusive	4	Inconclusive	7
DARK	19	Negative	6	Inconclusive	4	Inconclusive	7
SATURATED	11	Positive	5	High	3	Inconclusive	4
DESATURATED	11	Inconclusive	5	Inconclusive	3	Inconclusive	4

**Fig. 4:**
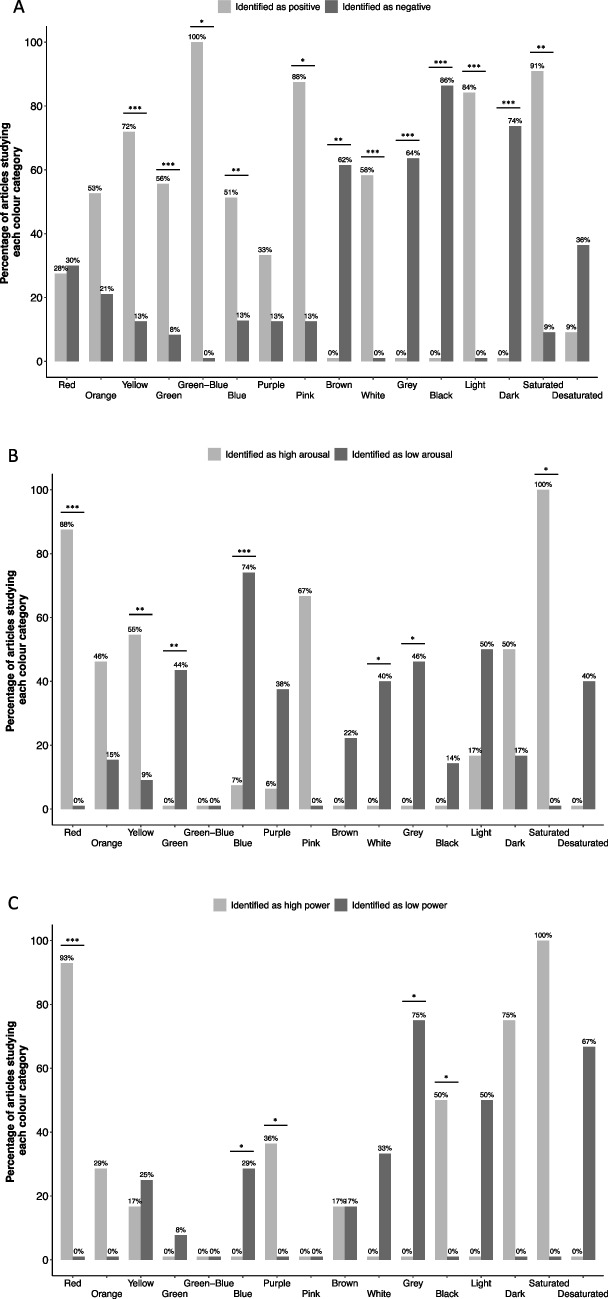
Colour correspondences with affective dimensions. A percentage of articles finding a correspondence between (**A**) valence (i.e., positive, negative), (**B**) arousal (high, low), and (**C**) power (high, low) and each colour category. See the number of articles corresponding to 100% for each colour category in Table 5. Significance from the chi-square tests coded as *p ≤ 0.050, **p ≤ 0.010, ***p ≤ 0.001. Note that we had low statistical power for some colour categories to detect differences due to a small number of articles. See supplemental material for studies reporting each correspondence

Here, we included articles using visual and verbal colour presentation modes and testing both explicit and implicit correspondences. The column “*n* of articles” refers to the number of articles that studied a correspondence between a particular affective dimension (e.g., valence) and a particular colour category (e.g., RED). Direction refers to statistically significant results, also marked with stars (e.g., ***) in Fig. 4. The term “inconclusive” refers to correspondences that could not be discerned statistically. The trends of such results are visible in Fig. 4. See supplemental material for articles included in each count

**Table 6:** The number of articles reporting a correspondence between discrete affective terms and red, orange, or yellow

RED			ORANGE			YELLOW		
Affective terms	*n*	% from total articles (*n* = 54)	Affective terms	*n*	% from total articles (*n* = 17)	Affective terms	*n*	% from total articles (*n* = 41)
Anger/angry/rage/ fury/enraged/mad/ irritated/frustrated	38	70.37	Happy/happiness/joy/ joyful/jovial/merry/ cheerful	14	76.47	Happy/happiness/joy/ joyful/jovial/merry/ cheerful/cheery/smiley	37	90.24
Happy/happiness/joy/ joyful/jovial/merry/ cheerful	15	27.78	Amusement/fun	6	29.41	Pleasure/pleasant/ pleased/contentment	6	14.63
Love/affection	14	25.93	Pleasure/ pleased/ contentment	5	29.41	Exciting/enthusiasm/ stimulating/energetic/energized	6	14.63
Exciting/excitement/ enthusiasm/stimulating	6	11.11	Exciting/enthusiasm/ stimulating/energetic	5	29.41	Amusement/fun	5	12.20
Passion/lust	6	11.11	Surprise/surprised	3	17.65	Surprise/surprised/ astonished	5	12.20
Fear/fright/afraid/scared/terrified/panic	5	9.26	Angry	2	11.76	Fear/fright/scared/ mortification	3	7.32
Hostile	4	7.41	Interest	2	11.76	Admiration	2	4.88
Masterful	4	7.41	Active	1	5.88	Anger/angry	2	4.88
Pleasure/contentment	4	7.41	Admiration	1	5.88	Coward/cowardice	2	4.88
Powerful/strong	4	7.41	Anticipation	1	5.88	Envy/jealousy	2	4.88
Surprise/surprised	4	7.41	Carefree	1	5.88	Hope	2	4.88
Anxious/anxiety/nervous	3	5.56	Defiant/contrary	1	5.88	Anticipation	1	2.44
Defiant/contrary	3	5.56	Distressed/upset	1	5.88	Carefree	1	2.44
Hate	3	5.56	Disturbed	1	5.88	Disgust	1	2.44
Shame/shamed	3	5.56	Hope	1	5.88	Inspired	1	2.44
Active	2	3.70	Stressful	1	5.88	Interest	1	2.44
Admiration/admired	2	3.70				Kind	1	2.44
Amusement/fun	2	3.70				Lively	1	2.44
Brave/courage	2	3.70				Sadness	1	2.44
Disgust/disgusted	2	3.70				Triumphant	1	2.44
Embarrassed/embarrassment	2	3.70				Worry/chagrin	1	2.44
Evil/cruel/dreadful	2	3.70						
Guilt/guilty	2	3.70						
Jealousy	2	3.70						
Pride/proud	2	3.70						
Sadness/upset/misery	2	3.70						
Tense	2	3.70						
Agony/anguished	1	1.85						
Aroused	1	1.85						
Contempt	1	1.85						
Ecstasy	1	1.85						
Interest	1	1.85						
Regretful	1	1.85						
Romance	1	1.85						
Stressful	1	1.85						
Triumphant	1	1.85						
Troubled	1	1.85						
Vibrant	1	1.85						

See supplemental material for studies reporting each correspondence

**Table 7:** The number of articles reporting a correspondence between discrete affective terms and green, green–blue, or blue

GREEN			GREEN–BLUE			BLUE		
Affective terms	*n*	% from total articles (*n* = 35)	Affective terms	*n*	% from total articles (*n* = 8)	Affective terms	*n*	% from total articles (*n* = 40)
Relaxed/relaxing/relaxation/ peace/peaceful/serene/ quietness/soothing/soothed/ calm/calmness/calming	12	34.29	Pleasure/contentment	5	62.50	Sad/sadness/depression/ depressed/unhappy/ gloomy/sorrow	21	52.50
Happy/happiness/joy	7	20.00	Joy	4	50.00	Relaxed/relaxing/relaxation/peace/peaceful/serene/quietness/soothing/soothed/calm/calmness/calming	19	47.50
Envy/jealousy	6	17.14	Relief	4	50.00	Happy/happiness/joy/ joyful/elated/bliss	10	25.00
Comfortable/comfort	5	14.29	Admiration	1	12.50	Comfortable/comfort	7	17.50
Disgust/disgusted	5	14.29	Amusement	1	12.50	Pleasant/pleased/pleasure/ contentment	5	12.50
Pleasure/pleased/ contentment	5	14.29	Calmness	1	12.50	Relief	5	12.50
Excitement/exciting/ energized	3	8.57	Interest	1	12.50	Secure/safe	5	12.50
Fear/fearful	3	8.57	Refreshed	1	12.50	Tender/kind/gentle	5	12.50
Gentle/tender	3	8.57	Sad	1	12.50	Pride/proud	4	10.00
Hope	3	8.57				Hope/hopeful	3	7.50
Relief	3	8.57				Lonely/loneliness	3	7.50
Sadness/depressive/gloom	3	8.57				Admiration	2	5.00
Secure	3	8.57				Discouraged/defeat	2	5.00
Amusement	2	5.71				Fear/terror	2	5.00
Guilt/guilty	2	5.71				Interest	2	5.00
Interest	2	5.71				Amusement	1	2.50
Admiration	1	2.86				Compassion	1	2.50
Anger	1	2.86				Defending	1	2.50
Anxious	1	2.86				Disgust	1	2.50
Carefree	1	2.86				Envy	1	2.50
Compassion	1	2.86				Grateful	1	2.50
Embarrassed	1	2.86				Moody	1	2.50
Greed	1	2.86				Regret	1	2.50
Healthy	1	2.86				Satisfied	1	2.50
Pride	1	2.86				Strong	1	2.50
Stable	1	2.86				Tired	1	2.50
Strong	1	2.86				Trust	1	2.50
						Worry	1	2.50

See supplemental material for studies reporting each correspondence

**Table 8:** The number of articles reporting a correspondence between discrete affective terms and purple, pink, or brown

PURPLE			PINK			BROWN		
Affective terms	*n*	% from total articles (*n* = 20)	Affective terms	*n*	% from total articles (*n* = 16)	Affective terms	*n*	% from total articles (*n* = 13)
Sadness/depression/unhappy/ melancholy/dejected	6	30.00	Love/affection/eros	11	68.75	Disgust/disgusted	6	46.15
Pride/proud	5	25.00	Happy/happiness/joy/ cheerful/bliss	10	62.50	Sad/depressed/ Gloomy/unhappy/ melancholy/dejected	4	30.77
Calmness/calming/ relaxation/soothed	3	15.00	Pleasure/contentment/ delight	5	31.25	Bored/boredom	2	15.38
Fear/fright	3	15.00	Amusement	3	18.75	Anger	1	7.69
Love	3	15.00	Excitement/enthusiasm	2	12.50	Comfortable	1	7.69
Powerful/power/strong	3	15.00	Romantic/romance	2	12.50	Contempt	1	7.69
Anger/rage	2	10.00	Admiration	1	6.25	Contentment	1	7.69
Anxious/worry	2	10.00	Embarrassment	1	6.25	Disappointment	1	7.69
Envy/jealousy	2	10.00	Interest	1	6.25	Dull	1	7.69
Excitement/enthusiasm	2	10.00	Kind	1	6.25	Masterful	1	7.69
Happiness/merry	2	10.00	Pride	1	6.25	Pity	1	7.69
Masterful	2	10.00	Sadness	1	6.25	Powerful/strong	1	7.69
Pleasure/pleased/ contentment	2	10.00	Softness	1	6.25	Protective	1	7.69
Admiration	1	5.00				Regret	1	7.69
Boredom	1	5.00				Secure	1	7.69
Comfort	1	5.00				Shame	1	7.69
Compassion	1	5.00						
Despondent	1	5.00						
Disgust	1	5.00						
Embarrassment	1	5.00						
Fun	1	5.00						
Guilt	1	5.00						
Interest	1	5.00						
Passion	1	5.00						
Regal	1	5.00						
Regret	1	5.00						
Tiredness	1	5.00						

See supplemental material for studies reporting each correspondence

**Table 9:** The number of articles reporting a correspondence between discrete affective terms and white, grey, and black

WHITE			GREY			BLACK		
Affective terms	*n*	% from total articles (*n* = 23)	Affective terms	*n*	% from total articles (*n* = 20)	Affective terms	*n*	% from total articles (*n* = 28)
Relaxed/peace/peaceful/ soothing/serene/calm/ calmness/calming	8	34.78	Sad/sadness/depressed/ gloomy/unhappy/miserable/ melancholy/grief/dejected	15	75.00	Sad/sadness/depressed/ depression/unhappy/ upset/melancholy/misery/ sorrow/dejected	21	75.00
Happy/happiness/joy/ merry/elated	7	30.43	Fear/fright/terror	7	35.00	Fear/afraid/scared/ terrified/dreadful	19	67.86
Hope/hopeful	4	17.39	Bored/boredom/bleak	5	25.00	Anger/angry/rage/fury	11	39.29
Relief	4	17.39	Disappointment	5	25.00	Hate/hatred	6	21.43
Anger/rage/fury	3	13.04	Regret	3	15.00	Evil/malice/cruel/ despondent	6	21.43
Surprise/shock/ astonished	3	13.04	Tired/tiredness/exhaustion	3	15.00	Power/powerful/strong	5	17.86
Admiration	2	8.70	Anger/fury	2	10.00	Guilt/guilty	4	14.29
Bored/boredom	2	8.70	Disgust	2	10.00	Distressed	3	10.71
Emptiness	2	8.70	Guilt	2	10.00	Hostile	3	10.71
Fear	2	8.70	Shame	2	10.00	Disappointment	3	10.71
Compassion	1	4.35	Anxiety	1	5.00	Regret	3	10.71
Contentment	1	4.35	Calmness	1	5.00	Masterful	3	10.71
Gentle	1	4.35	Confusion	1	5.00	Disturbed	3	10.71
Honesty	1	4.35	Contempt	1	5.00	Death/doom	2	7.14
Loneliness	1	4.35	Loneliness	1	5.00	Shame	2	7.14
Love	1	4.35	Remorse	1	5.00	Contempt	2	7.14
Pride	1	4.35				Defiant/contrary	2	7.14
Safe/secure	1	4.35				Agony	1	3.57
Tender	1	4.35				Aversion	1	3.57
						Disgust	1	3.57
						Secure	1	3.57
						Satisfied	1	3.57
						Envy/jealousy	1	3.57
						Helpless	1	3.57
						Courage	1	3.57
						Dull	1	3.57

See supplemental material for studies reporting each correspondence

**Table 10:** The number of articles reporting a correspondence between discrete affective terms and categories light/bright, dark, saturated, and desaturated

LIGHT/BRIGHT			DARK			SATURATED			DESATURATED		
Affective terms	*n*	% from total articles (*n* = 7)	Affective terms	*n*	% from total articles (*n* = 7)	Affective terms	*n*	% from total articles (*n* = 4)	Affective terms	*n*	% from total articles (*n* = 3)
Happy/joy/merry	5	71.43	Fear	4	57.14	Happy/joy	4	100.00	Sad/sadness	3	100.00
Calming/relaxation	2	28.57	Sad/sadness/ gloomy	4	57.14	Relaxation	1	25.00	Fear/fright	2	66.67
Fright	1	14.29	Angry/anger	3	42.86	Strong	1	25.00	Angry	1	33.33
Hope	1	14.29	Disgust	2	28.57				Weak	1	33.33
Love	1	14.29	Powerful/strong	2	28.57				Worried	1	33.33
Relieving	1	14.29	Worried	1	14.29						
Surprise	1	14.29									
Weak	1	14.29									

See supplemental material for studies reporting each correspondence

#### Colour correspondences with affective dimensions

**Fig. 5:**
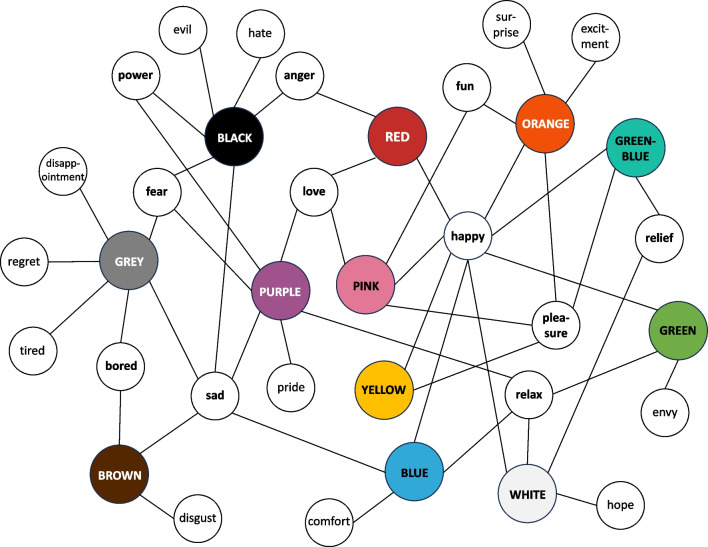
A visual representation of the most frequent correspondences between affective concepts and 12 colour categories, allowing to see the many-to-many correspondences. Each correspondence that was mentioned in at least 15% of articles (see Tables 6, 7, 8 and 9) is visualised here. The nodes are coloured for visualisation purposes only and distances between nodes have no significance. (Colour figure online)

There were 73 articles in total which we could use for colour correspondences with affective dimensions. As seen in Figs. 4 and 5 and Table 5, based on the chi-square tests, colour categories YELLOW, GREEN, GREEN–BLUE, BLUE, PINK as well as WHITE, LIGHT/BRIGHT and SATURATED were significantly more often reported to have positive correspondences while BROWN, GREY, BLACK, and DARK to have negative correspondences. A nearly identical number of articles reported RED to have positive and negative correspondences, making its valence ambivalent. When it came to arousal, statistically more articles reported colour categories RED, YELLOW, and SATURATED to have high arousal correspondences. Statistically more articles reported GREEN, BLUE, WHITE, and GREY to have low arousal correspondences. When it came to power, RED, PURPLE, and BLACK were statistically more frequently reported to have high power correspondences, while BLUE and GREY had low rather than high power correspondences. For the other colour categories, valence, arousal, and power differences could only be inferred qualitatively. Insufficient statistical power prevented us from detecting statistically significant differences (see trends in Fig. 4).

#### Colour correspondences with discrete affective terms

There were 61 articles in total on colour correspondences with discrete affective terms. In total, 190 different affective terms were used in the literature. Across all colour categories, these affective terms were used 1,032 times, with the most popular affective terms being *anger* (*n* = 48, 4.7% of instances), *sadness* (*n* = 46, 4.5%), *joy* (*n* = 46, 4.5%), *fear* (*n* = 46, 3.5%), *happy* (*n* = 32, 3.1%), *love* (*n* = 28, 2.7%), *pleasure* (*n* = 27, 2.6%), and *happiness* (*n* = 24, 2.3%; see Table A1 for all terms and their frequencies). Each colour category corresponded to several affective terms and those correspondences were not exclusive (many-to-many correspondences). As the diversity of affective terms was high, we merged synonymous terms when appropriate, and coined those *affective concepts* (e.g., types of sadness—*gloomy*, *depressed*, *unhappy*; types of boredom—*bored* and *boring*).

There were 23 frequent correspondences between colour categories and affective concepts, being mentioned in at least 15% of articles that considered a given colour category (see Fig. 5). Eleven of these correspondences were shared between several colour categories (highlighted in bold). The entire list of correspondences between affective terms and each colour category is reported in Tables 6, 7, 8, 9, 10 and include frequencies of each correspondence. In Tables 6, 7, 8, 9, 10, we always report all the terms extracted from the literature, and not just the affective concepts (e.g., *sad/sadness/depression/depressed/unhappy/gloomy/sorrow*).

## Discussion

*Colours are forces, radiant energies that affect us positively or negatively whether we are aware of it or not* (Itten, 1970, p. 12).

Interest in psychological, affective, and aesthetic effects of colour has a long history (for reviews, see Elliot, 2019; Evarts, 1919; Palmer, Schloss & Sammartino, 2013). While the experimental investigations into colour–emotion correspondences have been ongoing for over a century, there is a lack of systematic review of individual studies on the way colours link to emotions. To this end, we conducted a comprehensive systematic review on results from 132 empirical articles on colour–emotion correspondences. These articles were published between 1895 and 2022, reporting on a total of 42,266 participants from 64 different countries. Most articles had been published after 2010, though, indicating that the number of studies on colour–emotion correspondences has markedly increased.

By organising our results, we confirmed that researchers used different methodologies both in colour as well as emotion assessment. Thus, when presenting results, we could not account for each variation in method in isolation, and instead, collated observations (i.e., reviewed studies) across methods. For colour, irrespective of whether the authors worked with physical colours or colour terms, we focussed on the key colour categories, meaning the 11 basic colour categories (RED, ORANGE, YELLOW, GREEN, BLUE, PURPLE, PINK, BROWN, WHITE, GREY, and BLACK) as well as the regularly studied categories of GREEN–BLUE, LIGHT/BRIGHT, DARK, SATURATED, and DESATURATED.^4^ For emotion, we presented findings for affective dimensions (i.e., valence, arousal, and power/dominance) as well as for discrete affective terms (190 in total; e.g., *joy*, *anger*, *love*, *sadness*). We could not include results on the fourth affective dimension—novelty (Fontaine et al., 2007)—because no studies considered colour associations with this dimension. When making these groupings, we relied on the original authors’ decisions. That is, for each colour category, we followed the authors’ methods considering studies working with affective dimensions and/or discrete affective terms. As we followed the original authors’ approaches, we did not categorise discrete emotions along the affective dimensions ourselves (e.g. relabelling *joy* as positive).

We found systematic correspondences between colour categories and emotions, whether studies used affective dimensions or discrete affective terms. For instance, RED was linked to positive and negative, arousing, and high power emotions (e.g., *love, happiness, excitement, passion, anger, rage, fury, hostility, hate*). YELLOW and ORANGE were linked to positive and high arousal emotions (e.g., *happiness, pleasure, fun, excitement, surprise*). GREEN, BLUE, and BLUE-GREEN were linked to mainly positive, low arousal (i.e., calming) emotions (e.g., *comfort*, *happiness*, *relaxation*), but note that BLUE was also linked to *sadness* and GREEN to *envy/jealousy* in some studies. PINK was largely positive (e.g., *love*, *fun*, *happiness*), while PURPLE corresponded to some but not all empowering emotions (e.g., *pride*, *relaxation*, *love*, *fear*, *power*). WHITE was largely linked to positive and low arousal emotions (e.g., *happiness, relaxation,* *relief, hope*) while GREY and BLACK carried negative connotations, with GREY being more frequently linked to low power and low arousal (e.g., *fear, disappointment, regret, tiredness, boredom*), and BLACK to high power emotions (e.g., *fear, evil, hate, anger*). Worth noting, some colour–emotion correspondences were extremely frequent, being reported in the majority of studies. For instance, RED–*anger* correspondence had been observed in 73% of studies looking at the correspondences between RED and discrete affective terms. Other frequent correspondences were ORANGE–*joy* (76% of studies), YELLOW–*joy* (90% of studies), GREEN–BLUE–*contentment* and *joy* (63% and 50% of studies), BLUE–*sadness* (53% of studies), PINK–*love* and *joy* (69% and 63% of studies), GREY–*sadness* (75% of studies), and BLACK–*sadness* and *fear* (75% and 68% of studies).

Most colour–emotion correspondences were many-to-many, meaning that one colour category corresponded to several emotions, and one emotion, either as a discrete term or affective dimension, corresponded to several colour categories. In particular, *happiness* corresponded to eight colour categories (RED, ORANGE, YELLOW, GREEN, GREEN–BLUE, BLUE, PINK, and WHITE), *sadness*—to five colour categories (BLUE, PURPLE, BROWN, GREY, and BLACK), *pleasure*—to four colour categories (PINK, ORANGE, YELLOW, and GREEN–BLUE), *relaxation*—to four colour categories (BLUE, GREEN, PURPLE, and WHITE), *fear*—to three colour categories (PURPLE, GREY, and BLACK), *love*—to three colour categories (RED, PINK, PURPLE). Then, *boredom* (BROWN and GREY), *anger* (RED and BLACK), *power* (PURPLE and BLACK), *fun* (ORANGE and PINK), and *relief* (WHITE and GREEN–BLUE) each corresponded to two colour categories. Yet each colour category also had its own distinct pattern of emotion correspondences. For instance, only GREEN was linked to *envy*, WHITE to *hope*, PURPLE to *pride*, and BROWN to *disgust*. Diversity in colour meanings has been confirmed by independent studies showing that colour categories have wide associations, with participants naming many different concepts, objects, and ideas (Epicoco et al., 2024; Schloss, 2024; Tham et al., 2020). Potentially, this wide variety of associations contributes to shared affective meanings of colour (see Palmer & Schloss, 2010, for colour preferences). Then, some of these many-to-many colour–emotion correspondences might have been driven by cultural differences (e.g., RED being very positive in Chinese culture; Kawai et al., 2023). However, we cannot ignore the high comparability of colour–emotion correspondences in cross-cultural studies (Adams & Osgood, 1973; Jonauskaite, Abu-Akel, et al., 2020). Thus, other individual differences likely explain the observed variation in colour–emotion correspondences.

When considering colour dimensions (i.e., lightness, saturation, and hue), light colours had more positive connotations and dark colours had more negative connotations. This lightness-valence effect was strong and transcended colour categories, being true both when considering the achromatic colour categories as well as the chromatic ones. Regarding the achromatic colour categories, LIGHT/BRIGHT and WHITE colour categories were positive in all the studies, while DARK and BLACK colour categories were negative. Also, there was a higher affective similarity between GREY and BLACK than between GREY and WHITE, indicating that GREY could not be considered as an affectively neutral colour category. Instead, all reviewed studies reported it being negative. Regarding the chromatic colour categories, YELLOW, ORANGE, and PINK all carried exclusively positive connotations. They all covered a comparably lighter range in the colour space than their darker neighbours BROWN and RED. BLUE and PURPLE, both covering a wide range of light and dark shades in the colour space (Lindsey & Brown, 2021), carried some positive and some negative connotations (see in depth discussions on the meanings of BLUE and PURPLE in Epicoco et al., 2024; Shirai & Soshi, 2023; Uusküla et al., 2023).

In addition to lightness, saturation was also an important colour dimension. Saturated colours corresponded to positive, high arousal, and high power emotions, while desaturated colours corresponded to negative, low arousal, and low power emotions (for the importance of saturation/chroma, see further Pazda et al., 2024; Schloss et al., 2020). Some systematic affective correspondences also emerged for the third colour dimension—hue. To begin with, emotion correspondences were particularly similar for perceptually adjacent colour categories: (i) YELLOW and ORANGE, and (ii) BLUE, GREEN, and BLUE-GREEN. We interpret this perceptual adjacency in the context of ‘colour temperature’ (i.e., warm–cool colours),^5^ which is essentially a different way to conceptualise hue (see also a review on colour– temperature correspondences in Spence, 2020). We found that warm (YELLOW, PINK) and cool (GREEN, GREEN–BLUE, BLUE) colour categories corresponded to positive emotions, likely explained by the valence–lightness correspondence just discussed above. However, warm colour categories further corresponded to emotions of high arousal (RED, YELLOW, ORANGE) and high power (RED, PURPLE), while cool colour categories corresponded to emotions of low arousal (GREEN, BLUE) and low power (BLUE). Therefore, there was a mapping between perceived ‘colour temperature’ (hue) and its correspondence with arousal as well as power.

As the last major observation, cool colour categories (i.e., BLUE, GREEN–BLUE, GREEN), as a group, carried more similar affective meanings thtion correspondences are in line with previous studies showing congruency in affectivean warm colours, as a group (i.e., RED, ORANGE, YELLOW, BROWN, PINK, PURPLE). In other words, there was a greater affective differentiation within the warm than cool colours. In addition to the greater differentiation from the affective point of few, previous studies demonstrated such differentiation from the perceptual and linguistic points of view. Perceptually, one needs smaller physical distances between colour samples to perceive them as different when judging warm versus cool colour samples (MacAdam ellipses on the CIE *xy* colour space; MacAdam, 1942). Linguistically, all known languages have a greater number of basic colour categories designating warm versus cool colours (Conway et al., 2020, 2023; Gibson et al., 2017; Lindsey & Brown, 2006). Consequently, each warm colour category covers a smaller perceptual area than each cool colour category, with the colour terms GREEN and BLUE covering 50% of the entire colour space (Dodgson, 2019). Such disparities between the warm and cool colours likely reflect different communication needs (Conway et al., 2020; Twomey et al., 2021). Having more words for the warm than the cool colour area would indicate that there has been a greater demand for communication in the warm colour space. For instance, humans may have developed words for colours of objects that they needed to talk about, such as berries, flowers, fire, and animals. The same would not be true for background entities such as forest, grass, and sky. Here, we extend this reasoning on colour naming to our results on colour–emotion correspondences. In particular, based on our findings on arousal and power, there might be a higher communication need for warm colours, related to heightened readiness for action and social signalling (objects, health, sex, social status).

Overall, in this systematic review, we found systematic patterns in colour–emotion correspondences across 64 different countries, 128 years of investigation, and different colour and emotion assessment modes. Systematic colour–emotion correspondences are in line with previous studies showing congruency in affective colour connotations across (i) countries (Adams & Osgood, 1973; Jonauskaite, Abu-Akel, et al., 2020; Jonauskaite, 2024; Ou et al., 2018; Specker et al., 2018), (ii) age groups (Jonauskaite et al., 2024), (iii) historical time periods (i.e., the last 200 years; Guan et al., 2024), (iv) colour terms and colour patches (Jonauskaite, Camenzind, et al., 2021; Jonauskaite, Parraga, et al., 2020; Xu et al., 2024; cf. T. Wang et al., 2014), (v) emotion terms and facial expressions (Suk & Irtel, 2010; Takahashi & Kawabata, 2018), (vi) participants with and without colour vision deficiencies (Jonauskaite, Camenzind, et al., 2021; Sato & Inoue, 2016), and (vii) congenitally blind and sighted participants (Saysani et al., 2021). Being highly congruent across cultures and populations, colour–emotion correspondences should be an effective medium for communication (see a theoretical framework for broad colour–concept correspondences in Schloss, 2024).

Shared colour–emotion correspondences might be rooted in common human history, regularities in human languages and environments, and/or shared cognitive biases (e.g., see Jonauskaite, Abu-Akel, et al., 2020; Palmer & Schloss, 2010; Spence, 2011; Twomey et al., 2024). Beyond regularities in languages and environments, one might interpret such systematic results as evidence for a globalized world. Potentially, colour–emotion correspondences become increasingly more similar as people share more and more information globally via the Internet and other communication channels, possibly driven by global consumerism and marketing. To test the generalisability of our conclusions, especially the role of globalisation, one would need to gather data from small-scale societies (e.g., Davidoff et al., 1999; Davis et al., 2021; Groyecka et al., 2019; Sorokowski et al., 2014; Taylor et al., 2013).

## Limitations

We included only peer-reviewed literature, meaning that we did not account for unpublished data (e.g., non-peer-reviewed conference proceedings, bachelor’s, master’s, or PhD theses). Then, we focussed on studies published in English, likely implying an Anglo-centric bias (see more general discussion on the Anglo-centric bias in emotion literature in Wassmann, 2017; Wierzbicka, 2009). The reviewed studies had been conducted in 64 different countries using different languages. Finally, we pooled results across colour and emotion assessment modes, countries, and different time periods. Any further separations would have resulted in small sample sizes of eligible studies, making inferences at best tentative. Yet we provide all study details in supplemental material for interested readers.

Regarding the reviewed studies, we observed a high diversity of methodologies for colour as well as emotion presentation and assessment. Such a diversity obviously added noise to our results. For instance, only half of all studies used colour models that are perceptually accurate and reproducible, such as CIE *LAB*, CIE *LCh*, Munsell Color System, Natural Color System (Fairchild, 2013, 2015; Hunt & Pointer, 2011). Without using perceptually accurate models, one cannot know (and cannot reproduce) the colours participants saw (this limitation was previously highlighted in Elliot, 2019). Then, the concept of hue was often confounded with the concept of colour category. As a reminder, hues cut through the perceptual space, including all degrees of lightness and saturation, while colour categories include different degrees of lightness and saturation only if the naming does not change (i.e., BROWN and YELLOW are two different colour categories, yet, both have the same hue—yellow; also see Jonauskaite & Mohr, 2022). It is thus likely that colour samples varied across studies, even if they had been labelled in the same way. Some studies opted for focal colours as samples, solving the issue of colour naming. In these latter cases, hue effects were confounded with those of saturation and lightness (e.g., focal yellow is much lighter and more saturated than focal blue; Regier et al., 2005). Hence, some differences between the colour categories and the reviewed studies could be attributed to the differences between colour samples.

Regarding the diversity of emotion assessment modes, most studies opted for affective terms, ensuring a more straightforward comparison across studies. However, the affective terms were highly diverse (190 different terms) and not all of them were strictly emotion terms (see what makes a word a better representative of emotion in Ferré et al., 2024). Then, studies were conducted in different languages, raising the possibility that English translations did not capture the full meaning of the original affective terms (Jackson et al., 2019; Romney et al., 1997). Furthermore, few studies used emotion stimuli other than affective terms, such as induced emotions/moods or facial expressions, or tested *experienced* emotions rather than associations with emotions. Currently, we do not know whether, for example, systematic associations between the concepts of *yellow* and *joy* would also mean that one feels *happy* while looking at yellow. The few studies that tested colour effects on emotions have been inconclusive (Al-Ayash et al., 2016; Weijs et al., 2023; Wilms & Oberfeld, 2018).

Finally, in this systematic review, we only included studies working with context-free colour–emotion correspondences. Likely, various colour–emotion correspondences would be enhanced or changed when considered in specific contexts. For instance, red might become more negative in a combative context as opposed to a romantic one (e.g., Winskel et al., 2021), while green might become disgusting when seen on the surface of a milk product (i.e., a sign of mould). An empirical study, indeed, showed that participants’ preference for red could be temporarily (i) decreased by showing negative red images (e.g., blood) and (ii) increased by showing positive red images (e.g., berries; Strauss et al., 2013). These observations support the Colour-in-Context theory (Elliot & Maier, 2007, 2012; Meier et al., 2012), which states that relevant colour meanings are selected from the pool of all possible connotations based on the context in which the colour appears. These observations also support the Colour Inference Model (Schloss, 2024), which suggests that colour meanings are flexible and context dependent. The context-free colour–emotion correspondences reported in the current systematic review might constitute such a pool of available colour meanings. Moreover, not everybody shares the same colour–emotion correspondences (hence, many-to-many associations). This interindividual variation might emerge from individuals ideating different contexts or making different inferences.

## Conclusions

People systematically and reliably associate colours with emotions, as shown in 132 studies, spanning 128 years, and including over 40,000 participants. We found that the studied colour categories had distinctive patterns of emotion correspondences, often corresponding to several emotions (i.e., many-to-many correspondences). Approaching emotion both as dimensions and as discrete terms was fruitful, as each approach revealed slightly different affective colour connotations. Beyond individual categories, we observed systematic correspondences with lightness, saturation, and hue (‘colour temperature’). Lighter colours were linked to more positive emotions and vice versa. Then, more saturated colours were linked to more positive emotions of higher arousal and higher power. Finally, warm colours had more diverse emotion correspondences than cool colours, with warm colours representing more arousing and more powerful emotions. Overall, our results support the notion of widely shared colour–emotion correspondences (Adams & Osgood, 1973; Jonauskaite, Abu-Akel, et al., 2020). Differences in affective connotations of colours could be explained through different communication needs (Twomey et al., 2021), with warm colours being more pertinent to human survival, and so corresponding to more arousing and empowering emotions. Future studies should investigate whether these abstract colour–emotion correspondences translate to colour impact on experienced emotions, which is important for applied domains like design or health sectors (e.g., see Divers, 2023; O’Connor, 2011, 2023; Whitfield & Whelton, 2015). Thus, for now, we do not know if we *feel* colours, but we know that colours *convey* emotions.

## Supplementary Information

Below is the link to the electronic supplementary material.

Supplementary file1 (DOCX 1189 KB)

## Data Availability

The data are available online (https://osf.io/g5srf).

## Footnotes

1 https://www.verywellmind.com/color-psychology-2795824#toc-modern-research-on-color-psychology

2 https://www.cnbc.com/2021/09/10/paint-is-getting-costlier-and-harder-to-find-and-this-could-just-be-the-beginning.html

3 We could not include results on the fourth affective dimension—novelty (Fontaine et al., 2007), because no studies considered colour associations with this dimension.

4 We considered LIGHT/BRIGHT, DARK, SATURATED, and DESATURATED as categories and not continuous variables, because of the data we extracted. For instance, we extracted affective terms associated with LIGHT colours without further evaluating the criteria that authors used to determine which colours were light. Just like the other categories, diverse colour samples (and colour terms) were collated into the same category.

5 While there is some debate on what exactly constitutes warm and cool colours (Hardin, 2000), colour mixing theories and empirical studies agree that RED, ORANGE, and YELLOW are warm colours, while BLUE is a cool colour (Itten, 1961; Knoblauch et al., 2023; Newhall, 1941). PINK has a red hue, and BROWN has a yellow or orange hue, thus, they are considered warm as well. GREEN is often categorized as a cool colour (e.g., Holmes & Regier, 2017; Lindsey & Brown, 2006; Newhall, 1941), although, strictly speaking, this categorisation depends on the exact shade of GREEN. If green shades are closer to blue hues, they are cooler than those closer to yellow hues. Likewise, PURPLE is often rated as a warm colour (Knoblauch et al., 2023; Newhall, 1941), but it can be perceived as warmer or cooler, depending on whether it is perceptually closer to red or blue hues. Finally, Conway et al. (2020) suggested warm (RED, ORANGE, YELLOW, BROWN), cool (BLUE, GREEN), and intermediate (PURPLE, PINK) groups, based on their data on the communicative efficiency of colour naming.

## Code availability

Not applicable as no custom code was created for this study.
