# Supplementary material for: Do we feel colours? A systematic review of 128 years of psychological research linking colours and emotions
Source: Psychon Bull Rev. 2025 Jan 13;32(4):1457–86. doi: 10.3758/s13423-024-02615-z (PMC12325498; doi:10.3758/s13423-024-02615-z)
Supplement: Supplementary file 1 — Supplementary file1 (DOCX 1189 KB) [file 13423_2024_2615_MOESM1_ESM.docx]

# Appendix

Table A 1. All affective terms used in the reviewed articles, ordered by frequency.

| **Affective term** | **Count of instances** | **% from total instances** |
| --- | --- | --- |
| anger | 48 | 4.65 |
| sadness | 46 | 4.46 |
| joy | 46 | 4.46 |
| fear | 36 | 3.49 |
| happy | 32 | 3.10 |
| love | 28 | 2.71 |
| pleasure | 27 | 2.62 |
| happiness | 24 | 2.33 |
| sad | 18 | 1.74 |
| contentment | 18 | 1.74 |
| disgust | 18 | 1.74 |
| angry | 17 | 1.65 |
| amusement | 17 | 1.65 |
| relief | 16 | 1.55 |
| strong | 14 | 1.36 |
| cheerful | 14 | 1.36 |
| calmness | 13 | 1.26 |
| peaceful | 13 | 1.26 |
| admiration | 12 | 1.16 |
| calm | 12 | 1.16 |
| exciting | 12 | 1.16 |
| hope | 12 | 1.16 |
| comfortable | 11 | 1.07 |
| interest | 11 | 1.07 |
| powerful | 11 | 1.07 |
| pride | 10 | 0.97 |
| secure | 10 | 0.97 |
| masterful | 10 | 0.97 |
| joyful | 10 | 0.97 |
| unhappy | 9 | 0.87 |
| surprise | 9 | 0.87 |
| disappointment | 9 | 0.87 |
| jealousy | 9 | 0.87 |
| regret | 9 | 0.87 |
| envy | 8 | 0.78 |
| guilt | 8 | 0.78 |
| soothing | 8 | 0.78 |
| stimulating | 8 | 0.78 |
| hate | 8 | 0.78 |
| jovial | 7 | 0.68 |
| peace | 7 | 0.68 |
| passion | 7 | 0.68 |
| fright | 7 | 0.68 |
| shame | 7 | 0.68 |
| hostile | 7 | 0.68 |
| merry | 7 | 0.68 |
| depression | 6 | 0.58 |
| tender | 6 | 0.58 |
| serene | 6 | 0.58 |
| enthusiasm | 6 | 0.58 |
| upset | 6 | 0.58 |
| gloomy | 6 | 0.58 |
| rage | 6 | 0.58 |
| defiant | 6 | 0.58 |
| contrary | 6 | 0.58 |
| dejected | 6 | 0.58 |
| boredom | 6 | 0.58 |
| melancholy | 6 | 0.58 |
| pleased | 5 | 0.48 |
| fury | 5 | 0.48 |
| contempt | 5 | 0.48 |
| relaxation | 5 | 0.48 |
| surprised | 5 | 0.48 |
| relaxed | 5 | 0.48 |
| calming | 5 | 0.48 |
| excitement | 5 | 0.48 |
| depressed | 5 | 0.48 |
| despondent | 4 | 0.39 |
| compassion | 4 | 0.39 |
| disturbed | 4 | 0.39 |
| loneliness | 4 | 0.39 |
| distressed | 4 | 0.39 |
| proud | 4 | 0.39 |
| fun | 4 | 0.39 |
| relaxing | 4 | 0.39 |
| scared | 4 | 0.39 |
| anxious | 4 | 0.39 |
| gentle | 3 | 0.29 |
| soothed | 3 | 0.29 |
| bored | 3 | 0.29 |
| embarrassment | 3 | 0.29 |
| kind | 3 | 0.29 |
| worry | 3 | 0.29 |
| bliss | 3 | 0.29 |
| carefree | 3 | 0.29 |
| elated | 3 | 0.29 |
| power | 3 | 0.29 |
| sorrow | 3 | 0.29 |
| active | 3 | 0.29 |
| tense | 3 | 0.29 |
| disgusted | 3 | 0.29 |
| comfort | 3 | 0.29 |
| guilty | 3 | 0.29 |
| stressful | 2 | 0.19 |
| tiredness | 2 | 0.19 |
| anticipation | 2 | 0.19 |
| dreadful | 2 | 0.19 |
| worried | 2 | 0.19 |
| hopeful | 2 | 0.19 |
| embarrassed | 2 | 0.19 |
| romance | 2 | 0.19 |
| terror | 2 | 0.19 |
| energetic | 2 | 0.19 |
| courage | 2 | 0.19 |
| weak | 2 | 0.19 |
| astonished | 2 | 0.19 |
| safe | 2 | 0.19 |
| cruel | 2 | 0.19 |
| satisfied | 2 | 0.19 |
| misery | 2 | 0.19 |
| dull | 2 | 0.19 |
| terrified | 2 | 0.19 |
| pleasant | 2 | 0.19 |
| tired | 2 | 0.19 |
| energized | 2 | 0.19 |
| triumphant | 2 | 0.19 |
| emptiness | 2 | 0.19 |
| affection | 2 | 0.19 |
| miserable | 2 | 0.19 |
| cheer | 2 | 0.19 |
| afraid | 2 | 0.19 |
| evil | 2 | 0.19 |
| agony | 2 | 0.19 |
| vibrant | 1 | 0.10 |
| exhaustion | 1 | 0.10 |
| shock | 1 | 0.10 |
| mortification | 1 | 0.10 |
| coward | 1 | 0.10 |
| nervous | 1 | 0.10 |
| in love | 1 | 0.10 |
| panic | 1 | 0.10 |
| confusion | 1 | 0.10 |
| delight | 1 | 0.10 |
| suspicious | 1 | 0.10 |
| defeat | 1 | 0.10 |
| troubled | 1 | 0.10 |
| admired | 1 | 0.10 |
| moody | 1 | 0.10 |
| pity | 1 | 0.10 |
| cowardice | 1 | 0.10 |
| gloom | 1 | 0.10 |
| softness | 1 | 0.10 |
| defending | 1 | 0.10 |
| stable | 1 | 0.10 |
| grateful | 1 | 0.10 |
| brave | 1 | 0.10 |
| greed | 1 | 0.10 |
| fearful | 1 | 0.10 |
| chagrin | 1 | 0.10 |
| mad | 1 | 0.10 |
| grief | 1 | 0.10 |
| feeling secure | 1 | 0.10 |
| protective | 1 | 0.10 |
| frustrated | 1 | 0.10 |
| at peace | 1 | 0.10 |
| eros | 1 | 0.10 |
| quietness | 1 | 0.10 |
| inspired | 1 | 0.10 |
| enraged | 1 | 0.10 |
| shamed | 1 | 0.10 |
| refreshed | 1 | 0.10 |
| smiley | 1 | 0.10 |
| regal | 1 | 0.10 |
| irritated | 1 | 0.10 |
| aversion | 1 | 0.10 |
| ecstasy | 1 | 0.10 |
| regretful | 1 | 0.10 |
| aroused | 1 | 0.10 |
| being soothed | 1 | 0.10 |
| anguished | 1 | 0.10 |
| doom | 1 | 0.10 |
| death | 1 | 0.10 |
| hatred | 1 | 0.10 |
| lively | 1 | 0.10 |
| cheery | 1 | 0.10 |
| lonely | 1 | 0.10 |
| relieving | 1 | 0.10 |
| lust | 1 | 0.10 |
| remorse | 1 | 0.10 |
| malice | 1 | 0.10 |
| healthy | 1 | 0.10 |
| trust | 1 | 0.10 |
| romantic | 1 | 0.10 |
| friendly | 1 | 0.10 |
| helpless | 1 | 0.10 |
| discouraged | 1 | 0.10 |
| honesty | 1 | 0.10 |
| anxiety | 1 | 0.10 |
| bleak | 1 | 0.10 |
| depressive | 1 | 0.10 |
| TOTAL | 1032 | 100.00 |
